# Roles of the NFI/CTF gene family in transcription and development: update 2025

**DOI:** 10.20935/acadbiol8079

**Published:** 2025-12-25

**Authors:** Richard M. Gronostajski

**Affiliations:** 1 Department of Biochemistry and Program in Genetics, Genomics and Bioinformatics, Jacobs School of Medicine and Biomedical Sciences, University at Buffalo, Buffalo, NY, USA.

**Keywords:** nuclear factor I, nuclear factor one, development, transcription, gliogenesis, retina

## Abstract

This review is an update of the review entitled “Roles of the NFI/CTF gene family in transcription and development” published in *Gene* in 2000 (referred to hereafter as the 2000 *Gene* review). That review began with the sentence “The Nuclear Factor I (NFI) family of site-specific DNA-binding proteins (also known as CTF or CAAT box transcription factor) functions both in viral DNA replication and in the regulation of gene expression.”, which is as valid now as it was then. The subsequent focus of NFI research has been on the role of individual or multiple family members in gene expression during development, in various adult organ systems, and the effect of NFI mutations in human syndromes. Here we will cover post-2000 studies with an emphasis on the role of NFI genes in brain, skin, blood, skeletal muscle, and retinal development; recent studies on the effect of hemizygosity of NFI genes in human development; the role of NFI genes in cancer; and the recently described 3D structure of the NFIX DNA-binding domain.

## 1. Introduction

As described in detail in the 2000 *Gene* review [1], the NFI gene family was discovered as a required protein for the replication of Adenovirus type 2 and two years later identified as a site-specific DNA-binding transcription factor (TF) involved in the regulation of gene expression. We refer the reader to that review for information on the discovery of binding sites for NFI and the early characterization of NFI binding specificity, affinity, and homo- and heterodimerization of NFI family members. In addition, the 2000 review gives a detailed description of the structure of the NFI gene family in mammals and the single NFI genes in *Drosophila* and *Caenorhabditus elegans* [1]. Only minor additions have been found since then, primarily the discovery of additional alternative promoter regions with alternate 1st exons and numerous alternatively spliced isoforms of the four mammalian genes *NFIA*, *NFIB*, *NFIC*, and *NFIX*. There has been explosive growth in the number of papers published on the NFI gene family since 2000 (Figure 1), and this review will focus almost solely on work performed since then.

To summarize some of the most exciting discoveries regarding the NFI genes since the 2000 review: the use of knockout and/or conditional knockout alleles of the mouse NFI genes have shown how these genes are required for the development of multiple organ systems including brain, retina, muscle, liver, kidney, skin, lung, salivary gland and the hematopoietic and immune systems. Significant progress has also been made on the discovery of proteins with which NFI proteins interact to perform their functions. The role of NFI genes in various cancers has also been studied, as has the role of mutations in NFI genes in a number of rare genetic syndromes. A very large number of proteins have been shown to directly interact with various NFI family members in vivo. Finally, the structure of the NFIX DNA-binding domain has been determined, which may allow the development of therapeutics to treat diseases or syndromes in which NFI genes play a role. These and other findings are described in detail below.

## 2. Results

### 2.1. Use of germline knockout (KO) mice to study *NFI* gene function

While there was considerable circumstantial evidence that NFI proteins function in the regulation of developmental genes discussed in the 2000 *Gene* review [1], it was through the use of germline and conditional knockout (KO) mice that essential roles for specific NFI gene products have been determined. All of the germline and conditional deletions generated to date are deletions or disruptions of the 2nd exon of the NFI genes, which encode the bulk of the DNA-binding domain. The remainder of this section will describe the initial phenotypes seen in germline knockouts of the *Nfia, Nfib, Nfic*, and *Nfix* genes, in that order.

The first NFI gene to be disrupted was the mouse *Nfia* gene, whose homozygous germline disruption resulted in hydrocephalus, agenesis of the corpus callosum, aberrant hippocampus development, reduced GFAP expression, and significant perinatal lethality [2]. In addition, far fewer than expected heterozygous *Nfia*-disrupted mice survived to adulthood, indicating some degree of haploinsufficiency for *Nfia*. These findings will be discussed further in Sections 2.3 and 2.6 on “NFI genes in brain and spinal cord development” and “NFI genes in human syndromes, diseases and evolution”, respectively.

*Nfib* was the second NFI gene subjected to germline disruption by two groups, and homozygous germline loss of *Nfib* resulted in early perinatal lethality due to lung immaturity [3, 4]. In addition to the lethal lung defect, loss of *Nfib* resulted in fetal loss of the basilar pons, aberrant hippocampus development, reduced GFAP expression, and callosal agenesis [4, 5], and the latter three phenotypes were also seen in germline *Nfia*-knockout mice [2]. This was an early indication that *Nfia* and *Nfib* might share important functions in brain development. These data will be discussed further in Section 2.3 “NFI genes in brain and spinal cord development”.

Disruption of *Nfic* demonstrated that it is essential for normal tooth development in mice [6]. Homozygous loss of *Nfic* resulted in complete loss of molar tooth roots and aberrant incisor development due to defects in odontoblast development [7-9]. *Nfic* expression in tooth root formation appears due to a *Smad4*-*Shh*-*Nfic* signaling cascade required for root development [10]. *Nfic* was the first gene discovered whose disruption specifically affects tooth root development. These tooth defects cause postnatal lethality unless the mice are reared on a high-high-fat diet to allow nutrition sufficient for survival. Subsequent studies showed that *Nfic* is also required for normal bone development and adipocyte and osteoblast differentiation [11, 12], likely due to regulation of *Fgf* [13] and *Wnt* signaling [12]. *Nfic* also regulates the proliferation in dental epithelial, pulp, and bone marrow stem cells [14] and ameloblast differentiation [15]. *Nfic* appears to be a target of miR-184 since downregulation of this microRNA promotes osseous differentiation of periodontal ligament stem cells by upregulation of *Nfic* [16]. In addition, the compound suberoylanilide hydroxamic acid enhances odontoblast differentiation at least in part through increasing the expression of *Nfic* [17]. Some of these findings are reviewed here [18]. Lastly, *Nfic* has been linked to the regulation of wound healing through the *TGF-β1* pathway [19].

The first homozygous disruption of *Nfix* was postnatally lethal, with mice exhibiting hydrocephalus and partial agenesis of the corpus callosum [20]. In addition, spinal and bone ossification defects were seen [20]. A second germline KO of *Nfix* was generated from a conditional *Nfix* KO allele and found to have a similar but not identical phenotype [21]. In these animals, homozygous *Nfix* disruption resulted in delayed eyelid and ear opening and reduced weight gain, the latter of which could be partially alleviated with a soft dough diet [21]. Because these homozygous *Nfix* KO mice survived for a few weeks postnatally, their brains could be examined and were shown to be significantly larger than their WT littermates. This will be discussed further in Section 2.6 “NFI genes in human syndromes, diseases and evolution”. In addition, callosal and hippocampal dysgenesis and a reduced cerebellum were seen along with expansion of the cingulate cortex and excessive proliferation of *Dcx*+ cells in the lateral ventricles. *Nfix* expression was detected throughout telencephalon and hippocampus development from E12 to adult [21]. The cause of the differences in phenotype seen in the two KO mouse models is unclear, but could be due to differences in genetic backgrounds of the animals or due to differences in the KO allele, the first of which was a *lacZ* replacement of the second exon, while the second was a Cre-mediated deletion of the second exon. The first *Nfix* KO allele is no longer available (personal communication), while the second is now commercially available (JAX Strain #040481) and has been used for the studies discussed below.

### 2.2. Deletion of the single *NFI* gene in *C. elegans*

While there are four NFI family members in all examined mammals, there is only a single NFI gene in the nematode *C. elegans* (*nfi-1*) and in *Drosophila* [1]. Since NFI genes have been found in all the examined multicellular organisms but not in bacteria, yeast, or archaea [22], one question to be addressed was whether NFI genes are essential for multicellular development. Somewhat surprisingly, deletion of the DNA-binding domain of *nfi-1* resulted in relatively normal worm development but with locomotion defects, an egg-laying defect, a reduced adult life-span, and reduced pharyngeal pumping rate [23]. The egg-laying defect was seen most strongly in homozygous KO worms but was also present in heterozygous *nfi-1*−/+ worms, revealing an unusual haploinsufficiency in *C. elegans.* The pharyngeal pumping defect appears to be cell autonomous and connected in some way with the shortened lifespan in that rescue of both phenotypes can be seen in KO worms expressing *nfi-1* from the pharyngeal muscle-specific *myo-2* promoter [24]. The egg-laying and motility defects are not rescued in *myo-2::nfi*-1 worms. Thus, an NFI gene is not required for DNA replication or nearly normal development of *C. elegans*. The strong relationship between the worm *nfi-1* gene and the mammalian NFI genes was confirmed by demonstrating that the worm NFI-1 protein bound to the same consensus sequence as the mammalian NFI proteins [25]. Recently a chemical screen has been performed in *nfi-1*-deficient worms to identify possible therapeutic agents for Malan syndrome [26], an overgrowth condition [27] due to a mutation in a single copy of the human *NFIX* gene. This will be discussed further in Section 2.6 “NFI genes in human syndromes, diseases and evolution”. The *nfi-1* deficient worms are available from the Caenorhabditis Genetics Center (https://cgc.umn.edu).

### 2.3. *NFI* genes in brain and spinal cord development

#### Nfia:

Brain and spinal cord development is the area in which there have the greatest number of publications on the role of NFI genes. As described in Section 2.1, germline deletion of *Nfia* has dramatic effects on brain development [2], and many studies have extended these findings to determine the mechanisms leading to these defects. Homozygous disruption of *Nfia* was shown to disrupt the development of multiple glial structures during brain development, including the glial wedge, glial sling, and indusium griseum glia [28]. A frequently cited finding showed that *Nfia* controls the onset of gliogenesis in the chick and mouse spinal cord and is both necessary and sufficient for Glutamate-Aspartate Transporter protein induction in the developing ventricular zone (VZ) [29]. *Nfia* is also essential for the continued inhibition of neurogenesis in VZ progenitor cells [29], thus regulating the process known as the “gliogenic switch”. These findings indicated that *Nfia* was a master regulatory gene for gliogenesis in multiple regions of the brain and spinal cord. Subsequent studies showed that *Nfia* is expressed in many regions of the developing brain, including the prenatal deep cortical layers, subplate, and ventricular zone, and a large proportion of postnatal laterally projecting neurons [30]. These *Nfia* expression data, along with the mouse brain expression data for *Nfib* and *Nfix*, were subject to a review in 2009 [31]. Multiple lines of evidence indicate that *Nfia* is essential for normal gliogenesis, likely downstream of the Notch signaling pathway [32]. *Nfia* appears to bind to and promote the demethylation and activation of the *Gfap* gene in differentiating astrocytes [32], and binding sites of NFI proteins are also present in a number of other astrocytic genes, including *S100b*, *aquaporin4*, and *clusterin* [32]. In contrast, *Nfia* appears to function as a repressor of L1 gene expression in neuroblastoma cells [33], indicating that NFIA can function as either an activator or repressor of gene expression depending on the gene and cellular context. This concept is further supported by the finding that Nfia can repress expression of *Hes1* and thus downregulate the Notch signaling pathway in telencephalic progenitor cells [34].

The pathways through which *Nfia* induces gliogenesis are under active investigation. Sox9 and Nfia form a complex and coregulate the expression of a specific set of genes induced during gliogenesis [35]. *Lhx2* overexpression can override the activation of gliogenesis by both Notch signaling and *Nfia* overexpression, suggesting that *Lhx2* can antagonize *Nfia* activity in an as yet unknown manner [36]. *Pax6* overexpression can also override *Nfia*-induced astrogenesis in the developing hippocampus, suggesting that it acts similarly to *Lhx2* [37]. Neural stem cell models have proven useful in addressing *Nfia* function in astrogenesis. *Nfia* was shown to be a potent inducer of astrogenesis in mouse ESC-derived astrogenic neural progenitor cells (aNPCs) [38]. Here Nfia is bound to promoters and enhancers of multiple astrocytic genes and is associated with H3K27ac enrichment at these sites. Depletion of *Nfia* from early astrocytes in this model led to changes in astrocyte shape and a reduction in expression of *Gfap, Aldh1I1, Aldoc*, and *Aqp4*, along with an increase in neurogenic genes [38]. Nfia cooperates with Atf3 in binding to at least 47 promoter/enhancer sites during gliogenesis and increases chromatin accessibility as measured by ATAC-seq [38]. NFIA also induces gliogenesis in human long-term embryonic stem cell-derived neural stem cells, which spontaneously do not undergo the gliogenic switch [39]. This allows the production of large numbers of human astrocytes for future studies. Surprisingly, NFIA promotion of gliogenesis was most potent with a transient 5-10 day transgenic induction period followed by downregulation of NFI. Long-term induction of NFIA suppressed astrocytic differentiation. Transient NFIA induction also resulted in a lengthening of the G1 phase of the cell cycle, similar to what is seen during normal gliogenesis [39]. This suggests that continuous expression of NFIA might suppress gliogenesis by leading to G1 cell cycle arrest. TGFβ1-treatment of the neurogenic NSCs induced both NFIA expression and gliogenesis, but much less effectively than transgenic NFIA overexpression [39]. The potency of NFI genes for gliogenesis was shown most dramatically by the demonstration that a combination of NFIA, NFIB, and SOX9 induction was sufficient to reprogram mouse fibroblasts into astrocytes [40]. This direct trans-differentiation eliminates the need for embryonic or induced pluripotent stem cells, and if applicable to human cells, could be a source for patient-specific astrocytes. Along with its role in astrocyte development, loss of *Nfia* in adult mouse brains causes defects in astrocyte-mediated synaptic plasticity in the hippocampus, resulting in defects in learning and memory [41].

In addition to its role in gliogenesis, *Nfia* plays other roles in brain development. Conditional deletion of *Nfia* with a *Wnt1-Cre* allele eliminated the expression of *Nfia* in the dorsal root ganglion and resulted in a deficit in pinprick-responsiveness in the mice [42]; thus, *Nfia* appears necessary for the development of *Npy2r*-transient A-fiber nociceptor neurons. Germline disruption of *Nfia* greatly reduced cerebellar granule neuron (CGN) dendrite formation and the migration of cells from the cerebellum external granule layer [43]. Also, the expression of the cerebellar granule cell marker *Gabra6* was decreased ~4 fold in *Nfia* KO mice [44]. Indeed, transcriptome analysis of germline *Nfia* KO brains indicated that there was a general delay in the maturation of neurons and oligodendrocytes [45]. In addition, Nfia appears to provide a protective role in neurons as *Nfia*^*−*/*−*^ neurons are extremely sensitized to NMDA-induced excitotoxicity [46]. Nfia also inhibits the Sox10 induction of oligodendrocyte differentiation by forming a complex with Sox10 and preventing its function [47]. This inhibition of oligodendrocyte differentiation indicates a role for NFIA in suppression of oligodendrocyte maturation after white matter injury in hypoxia or multiple sclerosis [48]. With the availability of conditional *Nfia* KO alleles (JAX Strains #032281 and #040481), it is expected that the role of *Nfia* in neurons, oligodendrocytes, and other cell types in the brain will be examined in more detail in the future.

#### Nfib:

The sharing of some brain phenotypes between germline *Nfia* KO and *Nfib* KO mice (see Section 2.1) indicated that both genes were important in brain development. Further studies on the *Nfib* germline KO mice showed that a subpopulation of glia from the hippocampal ammonic neuroepithelium is lost, resulting in severe morphological defects [49]. *Nfib*^*−*/*−*^ brains have an increased number of proliferating cells in the cortical ventricular zone and increased levels of Ezh2 expression [50], and Nfib can bind to the promoter region of Ezh2 and repress its expression. Another mechanism by which Nfib may function is through forming a transcriptional regulatory network in adult neural progenitors with Sox11 and Pou3f4 to promote a neural fate [51]. There is a severe reduction in mature glial cells at the midline of the brains of *Nfib*^*−*/*−*^ mice with no changes in proliferation or cell death, indicating a severe defect in astrocyte differentiation [52]. It seems likely that the callosal agenesis or dysgenesis seen in these mice is due to these glial defects, as callosal axons do not appear to express *Nfib*. In addition to callosal defects, other forebrain commissures are aberrant in *Nfib*^*−*/*−*^ brains [52]. *Nfib*^*−*/*−*^ mice also exhibit a defect in EphrinB1 expression in the cerebellum [43], a phenotype shared with *Nfia*^*−*/*−*^ mice. As mentioned above, Nfib can cooperate with Nfia and Sox9 to directly convert fibroblasts to astrocytes, further suggesting an important role in gliogenesis [40]. One surprising finding is that Drosha targets *Nfib* in hippocampal neural stem cells and promotes neurogenesis, indicating that *Nfib* in these cells promotes oligodendrogenesis [53]. *Nfib* is also essential for the normal generation of corticofugal neurons throughout cortical development [54]. The availability of a conditional *Nfib* KO allele (JAX Strain #040481) will allow more detailed analysis of *Nfib* function in the brain.

#### Nfic:

While no brain defects were detected in germline homozygous *Nfic* KO mice [6], we describe in Section 2.6 “NFI genes in human syndromes, diseases and evolution” that *Nfic* may play a role in human brain development.

#### Nfix:

The expansion of the cingulate cortex and reduction in cerebellum size in germline *Nfix* KO brains indicated a significant role in brain development [21]. *Nfix* is expressed in the progenitor cells in the external granule layer of the cerebellum, and loss of *Nfix* delays the postnatal development of the cerebellar granule neurons, Purkinje cells, and Bergmann glia [55] along with an early postnatal decrease in proliferation in the granule cell layer. This identification of a role for *Nfix* in cerebellar granule neuron development is consistent with findings that expression of a dominant-repressor NFI-engrailed transgene in CGNs represses GABRA6 expression [44]. In the forebrain, Nfix is expressed in ventricular zone progenitor cells of the neocortex and hippocampus and appears to repress the expression of Bobby Sox (*Bbx*) in these cells, as deletion of *Nfix* increases *Bbx* expression [56]. This repression may be related to the finding that *Nfix* regulates neural progenitor differentiation in the developing hippocampus [57]. Homozygous deletion of *Nfix* alters the balance between self-renewal and differentiation of hippocampal neural progenitor cells, resulting in extended progenitor proliferation, distortion of the hippocampus, and decreased glial marker expression [57]. Nfix appears to interact with an alternatively spliced isoform of Lef1 to promote the quiescence of hippocampal NSCs [58]. Use of a conditional *Nfix* knockout allele showed that while loss of *Nfix* does not influence the survival of adult hippocampal neural stem cells, *Nfix* is required for the normal maturation and survival of neuroblasts derived from the stem cells [59]. These defects in hippocampal neuroblasts and neurons were associated with a deficit in learning tests in the adult mice [59]. Surprisingly, even heterozygous loss of *Nfix* is sufficient to induce abnormal hippocampus development and cause specific learning and memory deficits in adult mice [60]. These findings will be addressed further in Section 2.6 “NFI genes in human syndromes, diseases and evolution”. Some of these studies have been reviewed earlier [61].

Along with hippocampus defects, homozygous loss of *Nfix* causes major cortical defects. *Nfix*^*−*/*−*^ mice show increased levels of proliferation in the subventricular zone (SVZ) of the lateral ventricles with more Pax6-expressing progenitor cells [62]. Increased proliferation within the SVZ may be due to a role of *Nfix* in regulating neural stem cell quiescence [63]. In addition, the migration of neuroblasts through the rostral migratory stream (RMS) is reduced, and gliogenesis within the RMS is delayed in *Nfix*^*−*/*−*^ brains [62], leading to a smaller olfactory bulb. The defect in neuroblast migration occurs when *Nfix* is deleted from either adult neural stem cells in the SVZ or from migrating neuroblasts and appears related to aberrant neuroblast branching and increased expression of Npr2 in *Nfix*^*−*/*−*^ neuroblasts [64]. *Creb5* may promote the proliferation of SVZ neural stem cells through binding to the *Nfix* promoter and activation of its expression [65]. Surprisingly, deletion of *Nfix* in either adult hippocampal neural stem cells [59] or in cultured neural stem cells [66] causes a bias of differentiations towards the oligodendrocyte lineage, indicating that *Nfix* may normally repress oligodendrogenesis during gliogenesis. Finally, the hydrocephalus seen in *Nfix*^*−*/*−*^ brains appears due at least in part to aberrant ependymal cell development [67].

*Nfix* is also expressed in the developing spinal cord, and comparison of E14.5 Wild-Type and *Nfix*^*−*/*−*^ spinal cord showed 828 upregulated and 523 downregulated genes [68]. These changes are likely due to reduced gliogenesis in the spinal cords of *Nfix*^*−*/*−*^ mice [69].

A specific isoform of NFIX, NFIX-3, has been shown to be essential for activation of *GFAP* and *SPARCL1* in human astrocytes [70-73]. NFIX-3 was a much more potent activator of gene expression in transfected cells than the more common spliced isoforms NFIX-1 and NFIX-2. NFIX-3 is generated using an alternative promoter that is active in the nervous system [74]. This illustrates the likely importance of alternative promoter usage and splicing of NFIX isoforms in the induction of gliogenesis.

### 2.4. Redundancy and cooperation of *NFI* genes in neural development

The recent availability of a triple conditional KO mouse strain containing floxed alleles of *Nfia* [75], *Nfib* [76], and *Nfix* [21] (JAX Strain #040481) has made it possible to examine the role of these three NFI genes in development. However, the initial studies on NFI gene cooperation were performed using *Nfia* + *Nfib* floxed mice, which were available earlier from the authors’ laboratory. As discussed in Section 2.1, loss of either *Nfia* or *Nfib* results in significant brain defects [2-4]. However, these genes appear to cooperate strongly in brain development [77]. Homozygous KO of *Nfia* or *Nfib* results in the aberrant expression of 540 and 738 genes, respectively, while 91 of these genes are dysregulated in both KO brains [77]. Combined heterozygous loss of single *Nfia* and *Nfib* alleles yields a brain morphological phenotype similar to that seen with *Nfia*^*−*/*−*^ or *Nfib*^*−*/*−*^ animals, further suggesting a degree of redundancy between these genes. However, homozygous loss of both *Nfia* and *Nfib* yields a brain developmental phenotype significantly more severe than loss of either gene alone, showing cooperation between the 2 genes [77]. Cooperation between *Nfia* and *Nfib* was also seen with conditional deletion of the genes in neural stem cells in vitro [75]. Homozygous deletion of either *Nfia* or *Nfib* severely reduced astrocytic differentiation as expected, but had no effect on NSC proliferation. However, combined loss of both *Nfia* and *Nfib* resulted in cell cycle arrest of NSCs and complete loss of colony-forming ability [75]. This cooperation between *Nfia* and *Nfib* appeared specific since the combined loss of either *Nfia* and *Nfix*, or *Nfib* and *Nfix*, did not affect colony-forming ability. The overlapping but unique glial and neuronal expression patterns of NFI genes in adult cerebral cortex and hippocampus indicate multiple cell types in which NFI genes could cooperate [78].

Cooperation between NFI genes was also seen in the development of cerebellar granule neurons (CGNs). Homozygous loss of *Nfia* resulted in reduced Gabra6 expression in CGNs [44], as did homozygous loss of *Nfib* [43]. Moreover, multiple NFI proteins appear to occupy developmentally regulated genes in CGNs, again suggesting some degree of cooperation [79]. NFI genes have been implicated in Brain-derived neurotrophic factor-activation of CGN maturation by overcoming the repressive effects of *Nfatc4* from late CGN promoters [80]. ETV1 appears to cooperate with multiple Nfi proteins to promote late gene expression in CGNs [81]. *Nfix* is also expressed in the developing and adult cerebellum [82], and homozygous loss of *Nfix* results in a reduced number of CGN progenitor cells early in development but increased numbers by postnatal day 15 [83]. Finally, common regulatory targets of *Nfia*, *Nfib*, and *Nfix* have been identified during postnatal cerebellum development, suggesting cooperation between the 3 family members [84]. In spinal cord development, *Nfib* has been shown to regulate *Nfix* expression, which is essential for astrogenesis [69].

Another example of cooperation between three NFI genes is in the developing retina. Single-cell RNA-seq analysis performed on wild-type and various *Nfi*-deficient mice showed an additive effect of loss of *Nfi* genes on retina development [85]. *Nfia*, *Nfib*, and *Nfix* are all expressed at various levels in retinal progenitor cells, being more highly expressed at E18 and postnatally [85]. Conditional deletion of *Nfia*, *Nfib*, or *Nfix* alone results in minor but significant changes in retinal development, but combined deletion of *Nfia* and *Nfib* has a larger effect, reducing expression of several bipolar cell and Müller glial markers. Deletion of all three *Nfi* genes resulted in a near-complete loss of Müller glia and bipolar cells with significant disruption of retinal lamination [85]. Surprisingly, loss of all three *Nfi* genes increased proliferation of normally quiescent Müller glial cells in the postnatal retina, suggesting that *Nfi* genes regulate the differentiation and cell-cycle exit of retinal progenitor cells [85]. Combined inhibition of Notch signaling and deletion of *Nfia*, *Nfib*, and *Nfix* resulted in conversion of almost all Müller glia in the adult retina into neurons [86], again showing the importance of these three genes in production and maintenance of glia.

There have been a number of papers published on the role of *Nfi* genes in the olfactory epithelium (OE), some of which were discussed in the 2000 gene review [1]. In more recent studies, binding sites for Nfi proteins were identified in the promoter regions of several OE-expressed genes [87]. Using conditional KO alleles of *Nfia*, *Nfib*, and *Nfix*, it was shown that dorso-ventral gradients of *Nfi* gene expression in the OE play a direct role in the appropriate spatial selection of the ~2000 olfactory receptor genes in olfactory neurons [88]. Further studies are needed to determine the mechanism by which specific *Nfi* genes influence olfactory receptor selection.

Binding sites for NFI proteins have been found to be highly associated with sites occupied by the chromatin modifier DPF2-S (a component of the BAF complex) in NPCs, but not in ESCs, suggesting a unique interaction between NFI proteins and this alternatively spliced chromatin modifier in NPCs [89]. This indicates that NFI proteins may function partially through recruitment of chromatin-modifying proteins.

### 2.5. *Nfi* genes in extra-neural systems

#### 2.5.1. Skin

The roles of multiple *NFI* genes have been examined in skin development. Conditional deletion of *Nfib* in skin using either *Sox9-CreER* or *K15-CrePGR* resulted in enhanced melanocyte stem cell self-renewal through changes in *Edn2* activity, which was identified as a direct target of *Nfib* activation [90]. The authors concluded that *Nfib* governed epithelial-melanocyte stem cell behavior in developing and adult skin. Combined deletion of both *Nfib* and *Nfix* using *Sox9-CreER* resulted in severe hair loss (alopecia) through an alteration of hair follicle stem cell identity [91]. The authors determined that this change in stem cell identity was due to the loss of Nfib and Nfix binding to super-enhancers essential for hair follicle stem cell maintenance. Surprisingly, loss of Nfi proteins elicited the ectopic development of unwanted cell lineages, indicating that Nfi proteins were essential for normal lineage commitment through changes in chromatin accessibility. As mentioned in Section 2.1, *Nfic* also has a role in skin, being important for skin wound healing [19].

#### 2.5.2. Branched organs

While deletion of *Nfib* has major effects on brain development (see Section 2.1, 2.3 and 2.4), the perinatal lethality seen in *Nfib*^*−*/*−*^ mice is due to defects in lung maturation [3, 4]. When examined using a condition *Nfib* KO allele, it was shown that deletion of *Nfib* in lung mesenchyme, but not in lung epithelium, resulted in the perinatal lethal delayed maturation of the lung epithelium [76]. This suggests a defect in mesenchymal–epithelial interaction reminiscent of that seen with loss of *Nfib* in skin [90]. Nfi proteins have also been shown to directly activate Surfactant protein C expression in cooperation with thyroid transcription factor [92]. Since lung maturation is a major complication of premature birth, it is of interest that *Nfib* and glucocorticoid receptor coregulate an overlapping set of genes during lung maturation [93]. Surprisingly, heterozygous *Nfib*^*−*/+^ mice exhibit enhanced survival to hyperoxic conditions, suggesting some protective effect of the slightly delayed lung maturation seen in these animals [94].

Other branched organ systems are also affected by the loss of *Nfib*. *Nfib*^*−*/*−*^ mice exhibit significant defects in the development of the submandibular salivary gland with a failure of differentiation of the tubule cells [95]. In developing mammary gland, different *Nfi* proteins differentially activated or repressed the WAP promoter in cooperation with the glucocorticoid receptor and STAT5A, suggesting different roles for the different gene products [96, 97]. More recent studies have shown *Nfib* and *Stat5* co-regulate essential genetic programs in mammary gland development [98]. The splice variant NFI-C2 protein has been shown to activate the carboxyl ester lipase gene during mammary gland development [99], and a glycosylated form of Nfic appears to regulate the clusterin gene during mammary gland involution [100]. The role of NFI genes in the mammary gland was reviewed in 2003 [101]. In another branched organ, *Nfib* has been shown to regulate genes involved in prostate-specific gene expression and Prostatic Hyperplasia [102, 103]. Finally, though not traditionally considered a branched organ, homozygous loss of *Nfix* results in defective meiotic progression of spermatogonia in the mouse testis [104].

#### 2.5.3. Hematopoiesis, immune system, and adipocytes

There is considerable evidence for multiple roles of *Nfi* genes in hematopoiesis. Use of homozygous *Nfia* KO embryonal stem cells and blastocyst complementation showed that *Nfia* is essential for normal T-cell activation [105]. JC virus multiplication in human hematopoietic progenitor cells (HPC) requires *Nfix* [106]. *Nfix* expression drops upon HPC cell commitment to the B-cell lineage, and ectopic expression of *Nfix* blocks B-cell development [107].

*Nfia* regulates the cell fate pathway from HPCs towards erythropoiesis and granulopoiesis [108]. *Nfia* appears to function in a circuit with miRNA-223 to regulate granulopoiesis [109] and to promote the erythroid differentiation of CD34+ HPCs [110]. In contrast, *Nfix* appears to promote HPC survival [111] at least partially through regulation of *c-Mpl* [112]. Nfix and Pu.1 co-occupy a number of super-enhancers in HPCs to regulate the balance and lineage commitment of hematopoietic progenitors [113]. Finally, FAIRE analysis of 3T3-L1 preadipocyte differentiation combined with siRNA-mediated knockdown of NFIA and NFIB indicated that these NFI proteins play an important role in adipocyte differentiation [114].

#### 2.5.4. Muscle development

Using lineage-specific Cre and a mouse conditional *Nfix* KO allele, it was shown that muscle-specific ablation of *Nfix* prevents the embryonic to fetal switch in skeletal muscle [115]. This function of *Nfix* appears conserved in zebrafish, with an additional role of *Nfix* in slow muscle fiber formation [116]. At least one mechanism by which *Nfix* affects muscle development and regeneration is through repression of the Myostatin gene [117]. Therefore, mice lacking *Nfix* in adult skeletal muscle show delayed muscle regeneration upon injury. Also, *Nfix* appears necessary for *Sox6* to bind and activate myosin heavy chain 1 during fetal muscle development [118]. Surprisingly, silencing *Nfix* appears to rescue a mouse model of muscular dystrophy (MD) by delaying muscle regeneration, suggesting suppression of *Nfix* as a possible therapy for MD [119-122]. Along with a direct role in skeletal muscle cells, *Nfix* is expressed in macrophages in adult muscle, and loss of *Nfix* in macrophages results in defects in muscle regeneration [121]. Loss of *Nfix* also induces tachycardia in mice by increasing L-type calcium current in the sinus node by a yet unknown mechanism [123, 124]. The roles of *NFI* genes in mouse development are summarized in Table 1.

### 2.6. Role of *NFI* genes in human syndromes, diseases, and evolution

The first evidence that *NFI* genes played a role in human syndromes was the observation that *NFIA* haploinsufficiency was associated with human brain malformation and urinary tract defects [127]. Perhaps surprisingly, defects in ureter development of homozygous and heterozygous *Nfia* KO mice were detected *after* the initial observation of this phenotype in 5 patients with disruptions of *NFIA* [127]. Several subsequent groups observed that the 1p32-p31 deletion syndrome in humans, which contains *NFIA*, results in ventriculomegaly and corpus callosum hypogenesis, two phenotypes shared with germline *Nfia* KO mice [128-133]. More recent studies strongly support the model that *NFIA* is the critical gene in the phenotype of 1p32-p31 deletion syndrome [129, 130, 134-136]. This now-named *NFIA* syndrome [137], which includes intellectual disability, corpus callosum hypoplasia, macrocephaly, seizures, and renal defects, would be a strong candidate for therapy using *NFIA*-expressing AAV gene therapies. In addition, reduction in fetal *Nfia* expression has been detected after binge alcohol ingestion in mice, suggesting that reduction in *Nfia* may contribute to some aspects of fetal alcohol syndrome [138]. A genome-wide association study (GWAS) indicates that *NFIA* is a potential susceptibility gene for bipolar disorder [139]. A specific variant of an antisense transcript through the NFIA locus that is expressed in adipocytes correlated with body mass index in American Indians [140]. A specific NFIA allele has also been associated with asthma plus allergic rhinitis [141].

Several patients have been identified with an unnamed syndrome associated with heterozygosity in the *NFIB* gene [136, 142]. This syndrome shares some phenotypes with *NFIA* syndrome, including macrocephaly and intellectual disability [137]. While only around 20 patients with this syndrome have been identified worldwide, it is likely that there are many undiagnosed patients who will be identified as knowledge of the syndrome increases among clinicians. *NFIB* has also been associated with activation of the autism spectrum disorder A-C haplotype [143]. As with *NFIA* syndrome, the *NFIB* syndrome would be a candidate for gene therapy. A specific allele of *NFIB* has been associated with enhanced metabolism of certain psychiatric drugs [144], likely through affecting Cytochrome P450 (CYP) enzyme expression [145, 146]. Downregulation of NFIB, NFIC, or NFIX has been associated with decreased CYP expression in human liver cells [125], consistent with previous findings that an NFIA isoform could activate the expression of CYP2A3 [126]. Finally, a different specific allele of NFIB has been associated with susceptibility to sciatica in a Finnish population [147].

Heterozygous mutations in the *NFIX* gene can result in two clinically distinct syndromes, Marshall–Smith syndrome and Malan syndrome (formerly known as Sotos Syndrome 2) [26, 148, 149]. The types of mutations in *NFIX* determine which syndrome is present. The more clinically severe syndrome, Marshall–Smith, appears due to dominant-negative mutations predominantly outside of the DNA-binding domain of NFIX, which generate a mutant form of the NFIX that can interact with and inactivate wild-type NFIA, NFIB, NFIC, and NFIX proteins [26, 148-155]. These patients exhibit accelerated skeletal maturation, failure to thrive, intellectual disability, and abnormal facies [149]. This type of severe phenotype has been reproduced in a novel mouse model of Marshall–Smith syndrome [156].

Malan syndrome was identified as a unique syndrome in 2010, and a number of patients previously characterized as Sotos type 2 have been reclassified as patients with Malan syndrome [26, 154, 157-168]. The identification of patients with Malan Syndrome has been greatly aided by the establishment of the Malan Syndrome Foundation in 2015 (https://www.malansyndrome.org). This foundation has identified over 350 individuals around the world as of November 2024 with a genetically confirmed diagnosis of Malan Syndrome. These individuals exhibit prenatal and postnatal overgrowth, macrocephaly, advanced bone age with some skeletal defects including scoliosis, slender body habitus, some developmental delay and behavioral problems, and ocular findings [157, 169]. The foundation supports research into the causes and therapies for Malan syndrome, including the development of worm and mouse models for the syndrome [156, 170].

While not directly related to a human syndrome, NFIX has been linked to human speech development [171]. *NFIX* was one of several differentially methylated genes among 63 maps of ancient and present-day humans and 6 chimpanzees, suggesting that changes in *NFIX* methylation may be related to speech development [171]. It is unclear whether the speech delays seen in some patients with Malan syndrome are related to this potential role of *NFIX* in the evolution of human speech.

While no specific syndrome has been associated with heterozygosity for *Nfic*, at least 12 patients with mutations in *Nfic* have been identified in the last decade, as reported by GeneMatcher [172-174]. A consortium is being established specifically to investigate the phenotype(s) of patients with mutations in *Nfic* (https://ern-ithaca.eu/our-research-activities/calls-for-collaboration/clinical-and-molecular-delineation-of-the-new-nfic-related-disorder/). It will be important for the patients and their families that a peer-reviewed publication describing such patients is completed soon. An *NFIC* single-nucleotide polymorphism that interacts with the APOE epsilon4 allele in predisposition to Alzheimer’s disease was identified in a trans-ethnic GWAS [175].

### 2.7. Role of *NFI* genes in cancer

*Nfia* has been proposed as a tumor-promoting factor in glioblastoma through negative regulation of p53, p21, and PAI1 [176]. However, *Nfia* expression in astrocytomas is associated with improved survival [177]. In small cell lung cancer (SCLC), *NFIB* was shown to be frequently amplified and to drive metastasis by increasing chromatin accessibility at many intergenic regions [178]. NFIB overexpression cooperates with Rb/p53 deletion to stimulate metastasis in SCLC [179]. Conversely, NFIB is proposed to play a subtype-specific tumor-suppressor role in glioblastoma [180]. Yet, NFIB was shown to mediate BRN2-driven melanoma cell migration and invasion by promoting *EZH2* and reducing *MITF* expression [181]. These and other early findings on NFI genes in cancer are reviewed here [182] and here [183]. Subsequent studies showed that NFIX promotes lung cancer metastasis, and downregulation of NFIX results in aberrant expression of 18 genes in lung cancer cells [184]. However, downregulation of NFIX predicts poor prognosis in lung adenocarcinoma, but not in squamous cell carcinoma [185]. NFIX was also identified as a component of a novel NFIX-STAT6 gene fusion that is believed to play a role in solitary fibrous tumor, a rare soft-tissue sarcoma [186]. NFIX appears to function as a tumor suppressor in breast cancer, potentially by inducing a delay in the G2/M phase to reduce proliferation [187]. The downregulation of NFIX by miRNA647 and/or miRNA-1914 has been proposed to promote colorectal cancer progression [188]. NFIX has also been identified using spatial transcriptome analysis to be important for gastric cancer progression by regulating gastric cell stemness and proliferation [189]. Other proposed roles for NFIX in cancer were recently reviewed [190]. More recently, NFIC has been identified as a potential therapeutic target in small-cell lung cancer [191]. NFIB has also been found to be a recurrent translocation partner with MYB to carcinomas of the breast and head and neck [192]. In addition, NFIB has been found to cooperate with CARM1 and to be arginine-methylated by CARM1 during the induction of SCLC [193]. Both NFIA and NFIB have been identified as oncogenic drivers of SHH-either NFIA or NFIB greatly attenuates medulloblastoma cell growth and improves survival in mice injected with medulloblastoma cells [194]. Similarly, NFIB and NFIX were identified as important drivers of androgen-receptor-driven prostate cancer, with siRNA-mediated suppression of both genes resulting in substantially decreased Arv7 binding to chromatin [195]. This reduction in androgen receptor binding is proposed to be due to decreased chromatin accessibility in the absence of NFIB and NFIX. Given the sometimes contradictory and complex associations of NFI genes and various cancers, more clarification is needed on the specific mechanisms by which NFI genes influence cancer. Once this is performed, it is possible that modulation of NFI gene activity may be an appropriate therapy for some cancers. Table 2 summarizes the various roles of NFI genes in cancer.

### 2.8. Role of *NFI* genes in viral DNA replication and transcription

While NFI was first discovered as a protein required for adenovirus 2 replication (reviewed here [1]), surprisingly little is known of NFI gene function in the transcription and replication of other viruses. The expression of different NFI isoforms has been associated with the replication of JC virus in human hematopoietic progenitor cells [106] and brain progenitor cells [196-199]. *Nfix* expression levels correlate with JC virus replication in human astrocytes and progenitor cells [196]. Phorbol myristate acetate and interleukin-1 beta stimulation of JC virus replication in glial cells is proposed to be through upregulation of NFI proteins [198]. Given the association of JC virus replication with progressive multifocal leukoencephalopathy in immunosuppressed patients [197], a better understanding of the role of NFI genes in the replication of this virus would be useful. In addition, NFI proteins repress expression from the Bel1 response element present in the spuma retrovirus [200].

The most heavily studied role of NFI proteins in transcription is in the glucocorticoid hormone-dependent mouse mammary tumor virus long terminal repeat promoter (MMTV-LTR). The NFI binding site identified in the MMTV-LTR was reviewed previously [1], and we will address studies since 2000 below. NFI protein binding was shown to be required for hormone-dependent activation of MMTV on both transiently transfected and chromosomally integrated templates [201]. NFI binding was required for both the association of the BRG1 chromatin remodeling complex and GR binding to MMTV templates in vivo. Glucocorticoid receptor (GR) and NFI appear to bind cooperatively on the MMTV promoter, and GR activation resulted in a 50-fold increase in NFI binding to its site [202]. Concomitant binding of NFI and Oct1 to the MMTV promoter induces a partial nucleosome positioning even prior to GR activation of transcription in vivo [203]. NFI and progesterone receptor also synergize in vitro to promote nucleosome phasing on the MMTV promoter [204]. NFI binding to the MMTV promoter in vivo requires the BRG1 complex cooperating with GR [205]. NFI colocalizes with GR, SRC1, CBP, Ap-2, and BRG1 at an MMTV array in vivo [206]. An NFIA-KDM5b fusion protein has been used to direct demethylation of the MMTV array in vivo, resulting in a reduction in H3K27ac and reduced elongation by RNAP2 [207]. The observation that GR has a very short residence time at the MMTV promoter array in vivo (~10 s) [208-210] has led to a new model for TF activity through a “hit and run” mechanism. It will be of interest to determine whether NFI protein occupancy at the MMTV array shows the same dynamic nature as does GR occupancy and how it is affected by GR activation.

### 2.9. NFIB in licensing of DNA replication origins

As described above, germline deletion of *Nfia, Nfib, Nfic*, *or Nfix* does not appear to significantly affect DNA replication per se during mouse development [2-4, 6, 20, 21]. However, several studies have indicated that NFI proteins may influence cell cycle kinetics, cell quiescence, and terminal cell differentiation in specific tissues [5, 55, 62, 63, 90, 108, 113, 114]. The most direct data that NFI proteins may regulate some aspects of DNA replication is the finding that NFIB regulates replication origin licensing in U2OS osteosarcoma cells [211]. In these cancer cells, NFIB is directly associated with components of the DNA replication machinery, including components of the pre-replication complex present at origins of replication. NFIB was bound to over half of the same sites as Origin Recognition Complex 1 (ORC1), and depletion of NFIB reduced ORC1 binding at origins [211]. Depletion or overexpression of NFIB affected the timing of origin firing. The authors concluded that NFIB plays a significant role in the creation of higher-order chromatin structures associated with DNA replication based on changes in Micro-C contact maps in NFIB-depleted cells. NFIB was also shown to play a role in nucleosome eviction around replication origins in vivo and near NFI binding sites on purified nucleosomes in vitro. Since NFIB, NFIA, NFIC, and NFIX can compete with each other for binding to sites in vitro [1], it is possible that the other three NFI genes may also play a role in DNA replication.

### 2.10. NFI protein interactions with other transcription factors (TFs), chromatin modifiers, and histones

The first description of the NFIC protein C-terminal proline-rich transactivation domain binding to histone H3 was published in 1995 and referenced in the 2000 *Gene* review [1]. More recent studies have shown that two subdomains of this C-terminal domain can independently interact with histone H3 and can independently stimulate SV40 replication in vivo when fused to a GAL4 DNA-binding domain in a GAL4-driven in vivo SV40 replication assay [212]. Co-immunoprecipitation (co-IP) studies showed that NFI-X3 forms a complex with STAT3 in U373 and HEK cells [72]. C0-IP studies also demonstrated that NFIA and Sox9 form a complex in E12.5 spinal cord [35]. Similar Co-IP analysis showed that NFIA, NFIB, NFIC, and NFIX proteins can directly bind to the TF TTF-1 in co-transfection studies [92]. These proteins can also cooperate with TTF-1 in stimulating transcription of the SPC promoter in transfected cells in vivo, suggesting a functional interaction [92]. Yeast-two hybrid analysis showed that NFIX interacted with CGGBP1 and HMGN1 to mediate activation of the HSF1 promoter during heat shock [213]. NFIA, NFIB, NFIC, and NFIX were shown to interact with the TF FOXA1 by reciprocal Co-IP in transfected JEG-3 cells, while overexpression of these NFI proteins suppressed the FOXA1-mediated activation of the PSA promoter in transfection assays [103]. These interactions likely mediate the role of NFIB in Prostatic Hyperplasia described in Section 2.5.2 above. NFIX was shown to directly interact with PU.1 by Co-IP in transfected 293T cells in a study described in Section 2.5.3 above on the role of NFIX in hematopoietic progenitor cells [113].

While the biased screens described above have yielded important insights into NFI function in several systems, a number of unbiased screens have also found binding partners for NFI proteins. A proximity-dependent biotinylation (BioID) and affinity purification mass spectroscopy (AP-MS) screen identified 6703 and 1536 protein–protein interactions (PPIs), respectively [214]. Remarkably, of the 202 TF-TF interactions detected, 118 were interactions with NFI family members. This study yielded a large number of previously unknown interactions of NFI proteins with other TFs and other components of the transcription and chromatin modification machinery. Among the TF families shown to interact with NFI proteins are PAX, SOX, TLX, ELF, ETF, FOX, GATA, HNF, KLF, and LHX family members, and others [214]. Fifty-five TF interactions were detected with one or more NFIs, 53 interactions with NFIA, 37 with NFIB, 14 with NFIC, and 5 with NFIX. NFIX was in the top 10 for number of protein–protein interactors in the BioID experiment. These and many other interesting and potentially biologically significant interactions between NFI proteins and other TFs and components of the transcription and chromatin modification machinery are described in this ground-breaking study [214], but are too numerous to be detailed here. As expected, NFI proteins were also found to interact strongly with each other, as had been noted in several earlier papers and described in the 2000 *Gene* review [1]. Since these experiments were performed only in 293 cells, it seemed likely that other interaction partners of NFI might be detected in other cell types. Future studies will be needed to determine the functional significance of many of the NFI-interactions detected.

More recently, the NFI family was subjected to an even larger and more detailed interaction screen and ChIP-seq analysis in multiple cell lines [215]. This study characterized NFI family members as key TFs regulating gene expression. In this remarkably comprehensive study, NFIA was found to have high confidence interactions (HCI) with 602 proteins, 421 in HEK293 cells, 53 in both HEK293 and U241 cells, and 75 in U251 cells alone (see Figure 6 of [215]). These findings indicate that the NFI proteins likely have highly cell-specific functional mechanisms of action. The four NFI proteins in total showed interactions with 17,279 proteins, which, when filtered, yielded 1752 HCIs with proteins required for mRNA processing, chromatin organization, transcriptional regulation, and other cellular functions. A total of 309 of these HCIs were shared by all NFI family members; however, several of the interactions were family-member-specific, suggesting possible specificity of action for individual family members even with a single cell type. Genome-wide ChIP-seq analysis of the four NFI isoforms showed over 55,000 binding sites for each, of which 15,879 were shared by all four family members. However, NFIA showed 25,008 unique binding sites while NFIB, NFIC, and NFIX exhibited 15,914, 19,221, and 36,163 unique sites, respectively. This dataset contained several previously described binding sites for NFI proteins, supporting the likely biological significance of the newly determined sites. The authors also provided detailed GO analyses for each of the four NFI isoform interaction partners and likely downstream targets, providing a rich and useful resource for future studies.

The authors also determined the interaction partners of NFIB4, a naturally occurring isoform of NFIB lacking the N-terminal DNA-binding domain [216]. As expected, NFIB4 showed no DNA-binding in ChIP-seq pulldowns; however, in proximity labeling interaction studies, NFIB4 showed cell-type-specific interactions with many proteins, most of which were not identified as interaction partners with NFIB1, the isoform used in the ChIP-seq and interaction studies described above [215]. This finding indicates that alternative spliced isoforms of NFI genes may play unique roles in biology. Since all four NFI family members have multiple alternatively spliced isoforms, additional studies are needed to determine the possible biological roles of different NFI isoforms. This study provides an incredible resource with which to guide such future studies.

### 2.11. Structure of NFI DNA-binding domain

While bioinformatic analyses indicated that the NFI DNA-binding domain was very distantly related to the SMAD domain [217], it was clear from pre-2000 studies that these two domains differ in their binding specificity and affinity. It has taken over 35 years of attempts to obtain the 3D structure of an N-terminal NFI DNA-binding domain, that of NFIX [218]. This 3D structure determination, using both X-ray crystallography and cryo-EM, explains a number of previous observations regarding the DNA-binding domain, including the essential nature of three Cysteine residues shown previously to be required for DNA binding, the importance of an oxidation-sensitive Cysteine residue for DNA binding, the dimeric assembly of NFIs on DNA, and the effect of metal chelators on DNA binding. The identification of a Zn^2+^ binding site on each monomer was unexpected but explains much about the structure and stability of the DNA-binding domain [218]. The authors note that the ~85% sequence identity between the NFI DNA-binding domains of the four human NFI proteins indicates that this structure can likely be used to identify important residues in all of the NFI proteins. The structure also confirms the previous observation that NFI proteins contain a “MH1-like” fold that is related to, but not identical to, that seen in SMAD proteins [217]. While this structure is a breakthrough in our understanding of NFI proteins binding to DNA, there are still a few unanswered questions. Previous studies measured a DNA-binding affinity for in vivo produced NFI proteins from porcine liver of ~10^*−*11^ M (see references in [1]), while the recent study measured an affinity of ~10^*−*9^ M. There could be a number of reasons for this 100 fold difference, including in vivo modifications that could affect binding or differences in the percentage of active DNA-binding protein in different samples. However, it is also possible that a longer C-terminal extension on the NFIX DNA-binding domain could stabilize its DNA binding. In addition, the recent study indicated that the NFIX N-terminal domain formed dimers on DNA but was a monomer in solution, while previous studies indicated that longer forms of NFI formed dimers that were stable in solution in the absence of DNA, and that different length forms of NFIs needed to be co-translated in order to form heterodimers while the mere mixing of different translated isoforms did not allow heterodimerization (see references in [1]). These data may also suggest that some C-terminal extension on the NFIX DNA-binding domain could promote stable dimerization in the absence of DNA. It will also be important in the future to determine the structure of the C-terminal transactivation/repression domains of the four NFI proteins and how they interact with components of the transcription and chromatin modification apparatus. Despite these unanswered questions, or perhaps because of them, this structure determination represents an incredibly important advance in our understanding of NFI-DNA binding [218].

## 3. Discussion

Since the 2000 *Gene* review has been cited over 600 times (see Google Scholar), it has not been possible to cite every paper on the NFI gene family published since then. The author has therefore chosen to focus on what he has the most expertise in and will hopefully be most important in the future.

The availability of conditional KO alleles of *Nfia*, *Nfib*, and *Nfix* should greatly facilitate future studies on the redundancy and/or cooperation between these 3 genes. A conditional KO allele of *Nfic* would also be greatly desired to be able to determine the mechanism by which it affects odontogenesis, adipogenesis, osteogenesis, and other physiological functions described in Sections 2.1 and 2.7. Such an allele would also make it possible to assess the interactions between Nfic and the other Nfi proteins. These alleles will also allow the easy investigation of the role of single Nfi genes in virtually every cell type in the mouse, and give suitable Cre alleles. Given the evidence discussed above that alternatively spliced isoforms of the NFI family members may have unique biological roles, mechanisms to overexpress or repress specific NFI isoforms would be useful in determining their functions.

Developing mechanisms by which we could express the individual NFI genes in vivo in specific cell types of patients lacking one allele of the gene would be important in developing therapeutics for the human syndromes discussed in Section 2.6, including Malan syndrome, Marshall–Smith syndrome, NFIA syndrome, and the currently unnamed NFIB and NFIC syndromes. Alternatively, finding a way to increase expression from the single functional allele of the genes mutated in these syndromes back to wild-type levels may prove therapeutically useful.

The availability of conditional KO alleles for *Nfia*, *Nfib*, and *Nfix* should also make it possible to examine the role of Nfi genes in mammalian DNA replication by comparing the effects of single, double, and triple KOs of the genes in various cell types during development in adult tissues. For example, we now know that combined deletion of *Nfia* and *Nfib* results in cell-cycle arrest in NSCs [75]. A careful examination of the role of single and multiple Nfi genes in DNA replication in other cell types is clearly warranted.

Finally, the structural determination of the NFIX DNA-binding domain could lead to potential therapeutics in conditions where NFIX overexpression is deleterious. Further structural studies should allow the determination of precisely how NFI proteins interact with components of the transcription, chromatin modification, and DNA replication complexes.

Given that the genetic tools to investigate the role of *NFI* genes in the mouse have been available for only a short period, future studies should greatly expand our knowledge of the roles of *NFI* genes in mammalian biology. The next 25 years should be as exciting, if not more exciting, than the last 25.

## Figures and Tables

**Figure 1 •:**
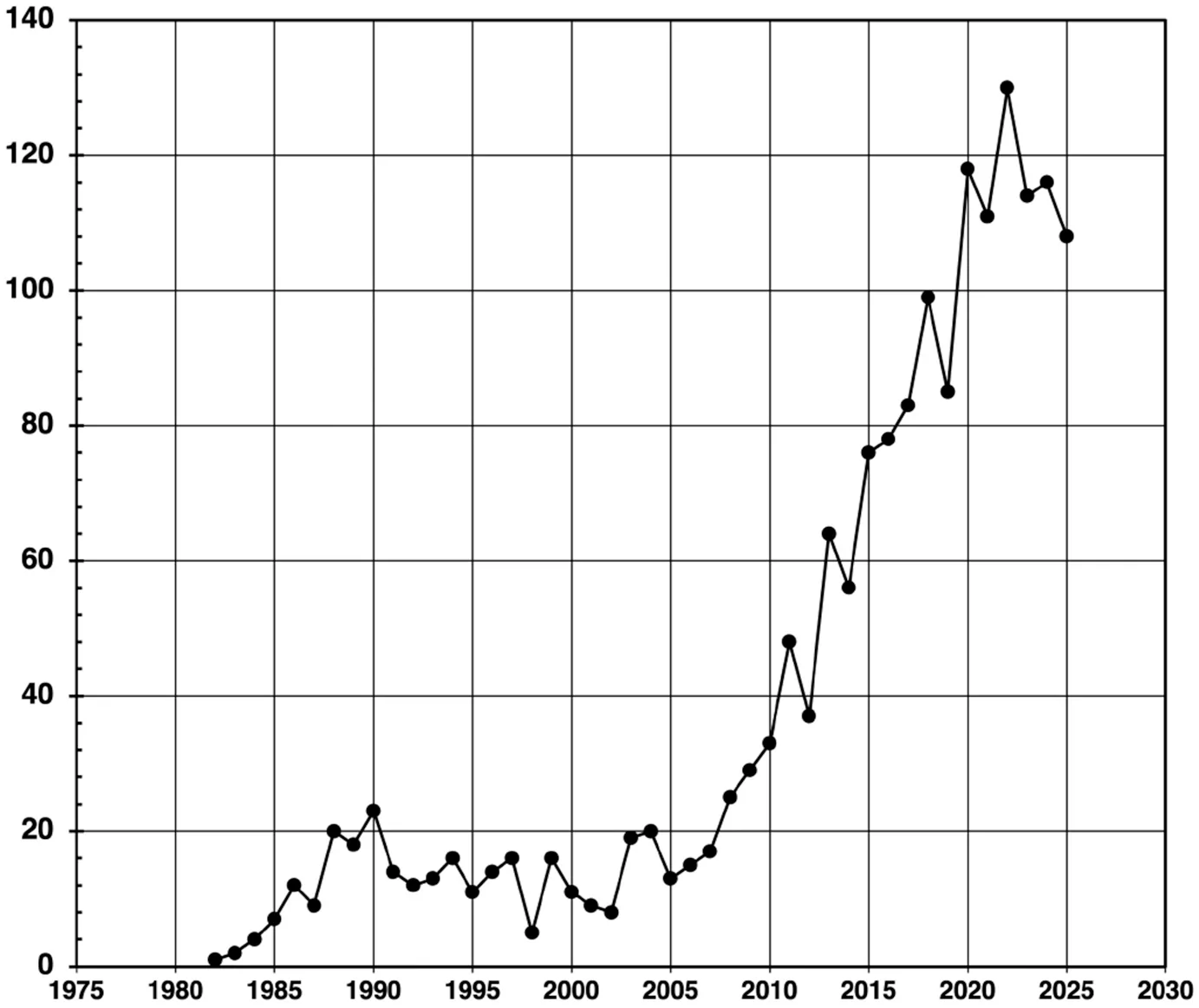
The graph shows the results of a search of Pubmed for the words “Nuclear Factor I”, or NFIA, NFIB, NFIC or NFIX. Note the rapid rise from ~2005 to the present. The decrease in 2025 may be due to the fact that the search was performed on 28 Oct 2025 missing 2 months of publication time in 2025. This search will miss some papers where the genes are incorrectly referred to as Nuclear Factor 1.

**Table 1 •:** Role of *NFI* genes in mouse development.

Organ or system	Gene	Roles	References
Brain	*Nfia*	Promote gliogenesis and neuron development	[2, 28-48, 77]
	*Nfib*	Promote gliogenesis and neuron development	[4, 5, 49-54, 77]
	*Nfix*	Promote gliogenesis and neuron development	[21, 55-67, 70-74, 82-84]
Spinal cord	*Nfia*	Promote gliogenesis	[29, 31]
	*Nfib*	Regulates *Nfix* expression	[69]
	*Nfix*	Promote gliogenesis	[68, 69]
Retina	*Nfia*	Promotes bipolar cell and Muller glia development	[85, 86]
	*Nfib*	Promotes bipolar cell and Muller glia development	[85, 86]
	*Nfix*	Promotes bipolar cell and Muller glia development	[85, 86]
Olfactory epithelium	*Nfia*	Gradients affect olfactory receptor gene selection	[88]
	*Nfib*	Gradients affect olfactory receptor gene selection	[88]
	*Nfix*	Gradients affect olfactory receptor gene selection	[88]
Skin	*Nfib*	Regulates melanocyte stem cell renewal	[90, 91]
	*Nfix*	With *Nfib* regulates hair follicle stem cell identity	[91]
	*Nfic*	Important for skin wound healing	[19]
Lung	*Nfib*	Essential in mesenchyme for late fetal lung maturation	[3, 4, 76, 92-94]
Salivary gland	*Nfib*	Required for gland and tubule cell maturation	[95]
Mammary gland	*Nfib*	Activates WAP promoter expression	[96-98, 101]
	*Nfic*	Activates WAP promoter expression	[99-101]
	*Nfix*	Activates WAP promoter expression	[96, 101]
Prostate gland	*Nfib*	Promotes hyperplasia	[102, 103]
Testis	*Nfix*	Required for proper spermatogonia meiotic progression	[104]
Hematopoiesis	*Nfia*	Required for T-cell activation, granulopoiesis, erythropoiesis	[105, 108-110]
	*Nfix*	Promotes JC virus replication in HPCs, HPC survival, HPC lineage commitment, suppresses B-cell development	[106, 107, 111-113]
Skeletal muscle	*Nfix*	Needed for embryonic to fetal fiber switch, loss attenuates MD	[115-122]
Cardiac muscle	*Nfix*	Needed for proper cardiac electrical signaling	[123, 124]
Liver	*Nfia*	Activation of *CYP2A3* expression, hepatocyte differentiation	[125, 126]
	*Nfib*	Activation of CYP expression, hepatocyte differentiation	[125]
	*Nfic*	Activation of CYP expression, hepatocyte differentiation	[125]
	*Nfix*	Activation of CYP expression, hepatocyte differentiation	[125]
Tooth	*Nfic*	Tooth root development	[6-10, 15, 18]
Bone	*Nfic*	Bone development	[11-14, 16, 18]

MD, Muscular Dystrophy; CYP, Cytochrome P-450.

**Table 2 •:** Role of *NFI* genes in Cancer.

Cancer	Gene	Roles	References
Glioblastoma	*NFIA*	Tumor Promoter by decreasing p53, p21 and PAI1 expression	[176]
	*NFIB*	Subtype-specific tumor suppressor	[180]
Astrocytoma	*NFIA*	Associated with improved survival	[177]
Small Cell (SC) lung	*NFIB*	Frequently amplified tumor promoter, increased chromatin access	[178, 179, 193]
	*NFIC*	Tumor promoter, identified as possible therapeutic target	[191]
NSC lung	*NFIX*	Stimulates cell migration and invasion but increases prognosis	[184, 185]
Melanoma	*NFIB*	Mediates cell migration and invasion, increases EZH2 expression	[181]
Fibrous tumor	*NFIX*	Fusion with STAT6	[186]
Breast cancer	*NFIX*	Tumor suppressor, induces delay at G2/M	[187]
Colorectal cancer	*NFIX*	Tumor suppressor	[188]
Gastric cancer	*NFIX*	Promotes stemness and proliferation	[189]
Meduloblastoma	*NFIB*	Tumor promoter	[194]
Prostate	*NFIB*	Promotes chromatin access and progression	[195]
Multiple types	*NFIB*	Fusion with MYB	[192]
Multiple types	All 4	Multiple functions reviewed	[182, 183, 190]

## Data Availability

No new data were created or analyzed in this study. Data sharing is not applicable to this article.
